# Comprehensive multiomics analysis of the effect of ginsenoside Rb1 on hyperlipidemia

**DOI:** 10.18632/aging.202728

**Published:** 2021-03-19

**Authors:** Jia Lianqun, Ju Xing, Ma Yixin, Chen Si, Lv Xiaoming, Song Nan, Sui Guoyuan, Cao Yuan, Yu Ning, Wu Yao, Zhao Na, Zhan Kaixuan, Yang Guanlin

**Affiliations:** 1 Key Laboratory of Ministry of Education for Traditional Chinese Medicine Viscera-State Theory and Applications, Liaoning University of Traditional Chinese Medicine, Shenyang, Liaoning, People's Republic of China

**Keywords:** ginsenoside Rb1, hyperlipidemia, microbiome, lipidomics, transcriptome

## Abstract

We analyzed the effects of ginsenoside Rb1 on hyperlipidemic in model mice. Using stool, plasma and hepatic tissue samples, we observed that the genera *Blautia* and *Allobaculum* were increased and *Turicibacter* was decrease in Rb1-treated mice as compared to untreated model mice. Ether lipid metabolism, glycerolipid metabolism, and glyoxylate and dicarboxylate metabolism were differentially enriched between the Rb1 and model groups. Lipidomics revealed 169 metabolites differentially expressed between the model and Rb1 groups in a positive ion model and 58 in a negative ion model. These metabolites mainly participate in glycerophospholipid, linoleic acid, and alpha-linolenic acid metabolism. The main metabolites enriched in these three pathways were phosphatidylcholine, diacylglycerol and ceramide, respectively. In a transcriptome analysis, 766 transcripts were differentially expressed between the Rb1 and model groups. KEGG analysis revealed lysine degradation, inositol phosphate metabolism, and glycerophospholipid metabolism to be the main enriched pathways. Multiomics analysis revealed glycerophospholipid metabolism to be a common pathway and phosphatidylcholine the main metabolite differentially enriched between the Rb1 and model groups. Results from fecal transplanted germ-free mice suggest that to suppress hyperlipidemia, Rb1 regulates gut microbiota by regulating the synthesis and decomposition of phosphatidylcholine in glycerophospholipid metabolism, which in turn decreases serum total cholesterol.

## INTRODUCTION

Cardiovascular disease (CVD) is the leading cause of death worldwide [1, 2]. Preventing hyperlipidemia reduces the likelihood that people will develop CVD. In that regard, ginsenosides, which are the major constituents of ginseng, have been reported to exert antihyperlipidemic effects [3]. Among them, protopanaxadiol (PPD) ginsenoside Rb1 is the most abundant saponin in ginseng and was recently found to significantly decrease plasma triglyceride levels and cholesterol and to increase the levels of total phospholipid, phosphatidylcholine and phosphatidylethanolamine in mice [4]. This suggests ginsenoside Rb1 could potentially play a preventive role in hyperlipidemia.

Microbial homeostasis plays an important role in regulating body health and preventing disease. Previous studies have confirmed that the composition of the gut microbiome is closely related to hyperlipidemia [5]. Moreover, the abundances of *Escherichia coli* and *Bifidobacterium* are reportedly increased in the intestinal microbial community of hamsters fed a high-fat diet [6], suggesting that a high-fat state has an important effect on intestinal microbial homeostasis. Another study found that *E. faecalis*, an intestinal probiotic, reduces the effects of cholesterol in hypercholesterolemic mice by activating the ABCG5 and ABCG8 transporters [7], which is consistent with there being a close relationship between intestinal flora and hyperlipidemia. In addition, increasing the relative abundance of beneficial bacteria (*Bifidobacterium* and *Lactobacillus spp.*) effectively prevented hyperlipidemia [8]. Thus, correcting balance of intestinal flora may be an effective method for preventing and treating hyperlipidemia.

Intestinal flora interacts with the host by activating signaling in the gut-brain axis, gut-lung axis, gut-liver axis and gut-renal axis [9, 10] Signaling in the gut-liver axis plays key roles in the regulation of lipid metabolism. On study showed that levels of 22 different lipids were abnormal in hyperlipidemic rats as compared to healthy rats [11]. This suggested that lipid metabolism plays an important role in the development of hyperlipidemia. When mice fed a high-fat diet were treated with hypolipidemic food, genes related to cholesterol metabolism (Nfil3, Nrep and Ldlrap1) and lipid storage (Mup5, Tuft1, and Lasp1) were significantly downregulated [12]. This suggests that expression of genes related to lipid metabolism play a significant role in reversing hyperlipidemia and that comprehensive analysis of the microbiome, lipidomics and transcriptome could potentially enable detection of the mechanism and treatments for hyperlipidemia.

Ginsenosides significantly alter the intestinal flora [13], and ginsenoside Rb1 also increases the level of LPCs and affects phosphatidylcholine metabolism in an Alzheimer’s disease mouse model [14]. However, the mechanism by which Rb1 relieves hyperlipidemia remains unclear. We therefore performed a comprehensive multiomics analysis to evaluate the microbiome, lipidome and transcriptome of hyperlipidemic mice to assess the effects of Rb1 in detail and shed light on the mechanism underlying Rb1’s antihyperlipidemic effect.

## RESULTS

### Confirmation of the successful construction of a hyperlipidemia mouse model

Compared with the control mice, the hyperlipidemic mice had significantly higher serum levels of triglyceride (TG), total cholesterol (TC) and low-density lipoprotein C (LDL-C), but significantly lower serum levels of high-density lipoprotein C (HDL-C). This indicates that our rat hyperlipidemic model was successful (Table 1 and Supplementary Figure 1A).

**Table 1:** Comparison of the serum lipid levels in mice from each group (mmol/l, Mean±SD, n=7).

**Group**	**TG**	**TC**	**HDL-C**	**LDL-C**
Control	0.60±0.07^#^	1.56±0.14^#^	1.23±0.15^#^	0.37±0.14^#^
Model	1.22±0.19^*^	2.06±0.20^*^	1.05±0.09^*^	0.63±0.08^*^
Rb1	0.89±0.10^#*^	1.74±0.11^#^	1.26±0.13^#^	0.48±0.06^#^

*: *P*<0.05 vs control; #: *P*<0.05 vs model.

### Microbiome sequencing characteristics and differential abundance of bacteria

**Figure 1:**
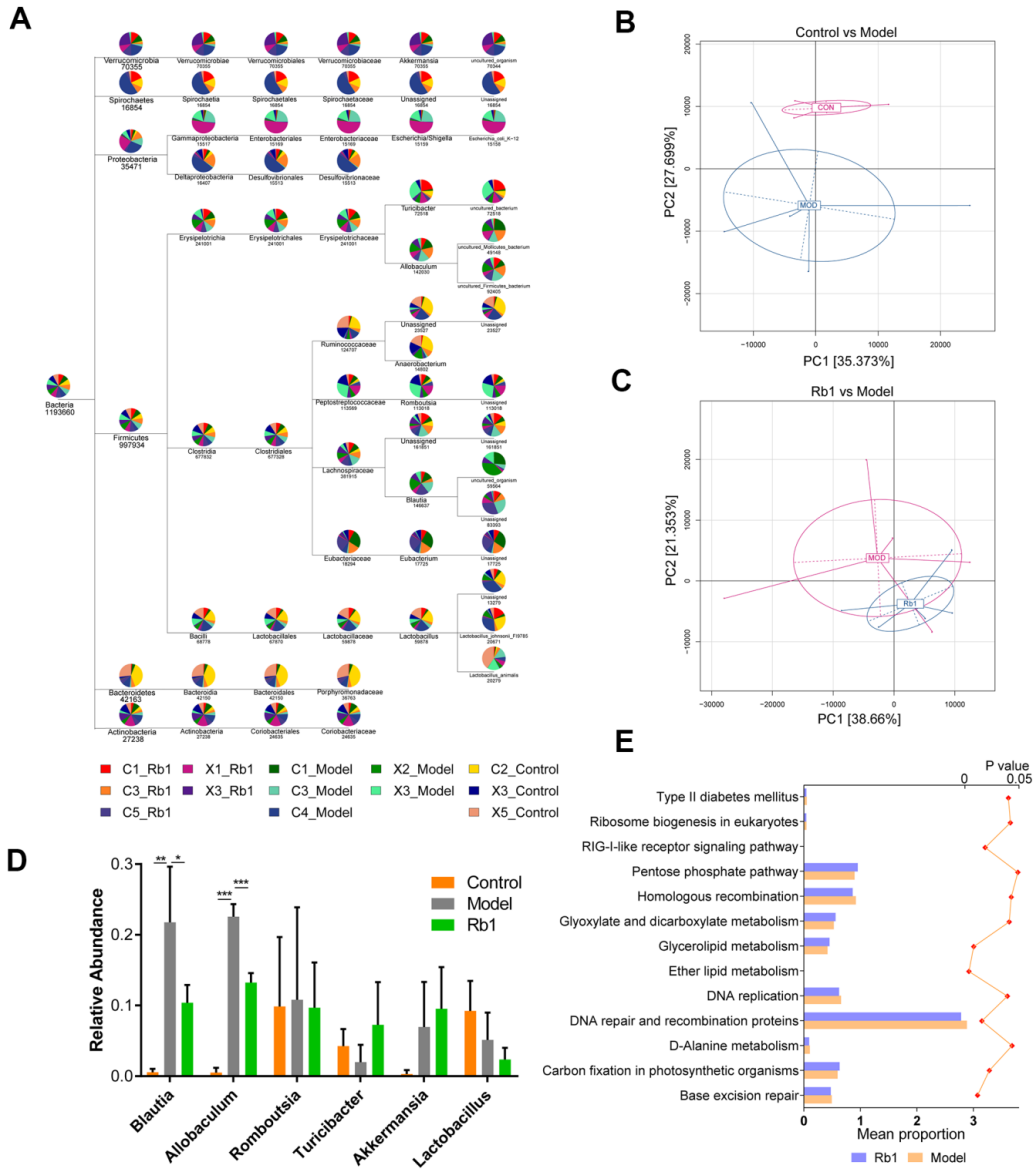
**Characteristics of 16S rRNA sequencing.** (**A**) TaxonTree revealed the distribution of each species in all samples. Each layer in the leaf node represents a classification level, from left to right is the boundary, gate, class, order, family, genus and species level, only genus annotated and with relative abundance large than 1% was shown in the figure. (**B**) PCA of OUT between Control and Model group. Each point in the graph represents a sample. The different colors of the points represent the grouping of the samples. (**C**) PCA of OUT between Rb1 and Model group. (**D**) Relative abundance of bacteria in genus level in each group. Blautia and Allobaculum increased in Model group and Turicibacter decreased in Model group. Notes: data was shown in mean +/- SD, * represent P<0.05, ** represent P<0.01, *** represent P<0.001. (**E**) KEGG analysis of abundance difference between Rb1 and model group. The result was performed by PICRUSt software with default parameters. Each color represents a group of samples, their average relative abundance is showed in X axis. The left of bar chart shows the pathways with significant differences in abundance between the two groups, and the right of chart shows P value of each pathway.

10,091,546 total sequences composed of 4,159,619,833 total bases and an average length of 412.19 bp were obtained from the optimized sequences. Based on these, 3730 operational taxonomic units (OTUs) were obtained with an average size of 27.14 organisms. After flattening for cluster analysis, 40 phyla, 72 classes, 82 orders, 153 families, 335 genus and 461 species were obtained (Figure 1A). The PCA plot intuitively displayed the similarities and differences between samples. The model and control groups were obviously discrete on the two-dimensional graph (Figure 1B), and the Rb1 and model groups were relatively discrete (Figure 1C). After statistical analysis of the annotated species, we found that at the genus level, the relative abundances of *Blautia* and *Allobaculum* were greater, and *Turicibacter* was lower, in the model group than the control group (Figure 1D). However, when Rb1 was fed to the model group, the relative abundance profile of these bacteria was reversed. KEGG analysis showed that the level of enrichment in ether lipid metabolism, glycerolipid metabolism, glyoxylate and dicarboxylate metabolism, and type II diabetes mellitus significantly differed between the Rb1 and model groups (Figure 1E).

### Characteristics of the lipidomics analysis and differential expression of lipids

**Figure 2:**
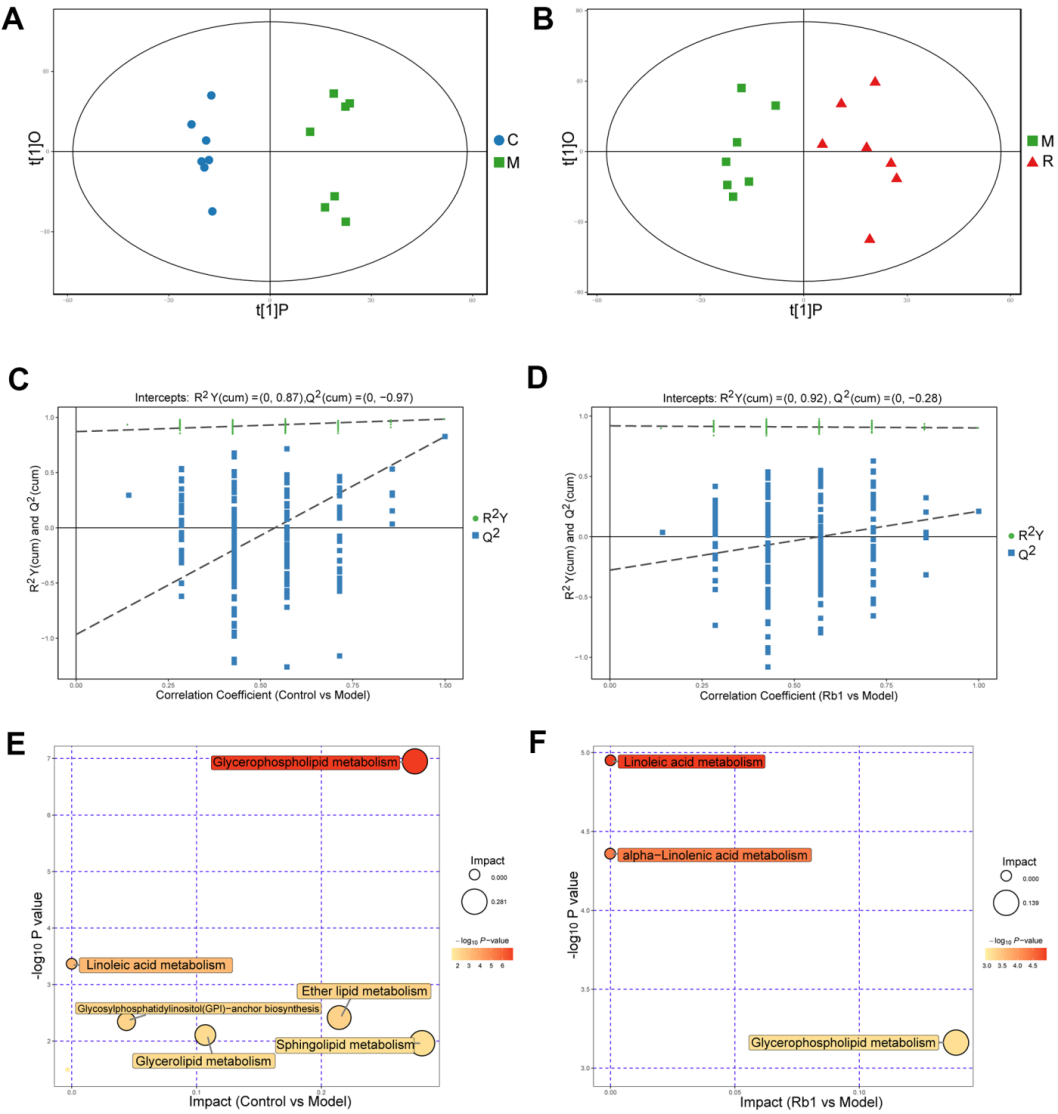
**Characteristics of lipidomics in positive ion model.** (**A**) Score scatter plot of OPLS-DA model for group C vs M in POS model. The X axis t [1] P denotes the predicted principal component score of the first principal component, the Y axis t [1] O denotes the orthogonal principal component scores. The two groups of samples are very distinct in this model. C, control group. M, model group. POS, positive ion model. (**B**) Score scatter plot of OPLS-DA model for group M vs R in POS. M, model group. R, Rb1 group. POS, positive ion model (**C**) Permutation test of OPLS-DA model for group Control vs Model in POS. The X axis represents the replacement retention, the Y axis represents the value of R^2^Y or Q^2^, the green dot represents the value of R^2^Y and the blue square represents the value of Q of the replacement test. The dotted lines represent the regression lines of R Y and Q, respectively. The original model can well explain the difference between the two groups of samples. (**D**) Permutation test of OPLS-DA model for group Rb1 vs Model in POS. (**E**) Pathway analysis for group Control vs Model in POS. The bubble size indicates the influencing factor in the topological analysis; the bubble color represents the P value of enrichment analysis. (**F**) Pathway analysis for group Rb1 ve Model in POS.

In positive ion mode (POS), a total of 4119 peaks were detected, 1891 metabolites were identified using the interquartile range denoising method, 815 metabolites significantly differed between the control and model group, and 169 metabolites were significantly differed between the model and Rb1 group. The differences between the control and model groups, between the model and Rb1 groups were distinct in the OPLS-DA plot (Figure 2A, 2B). The original permutation test for the OPLS-DA model explained the difference between the two compared groups of samples (Figure 2C, 2D). Using a criterion of P<0.05 in the enrichment analysis, KEGG and MetaboAnalyst revealed that different metabolites in comparison between the control and model groups mainly enriched in glycerophospholipid metabolism and linoleic acid metabolism pathways (Figure 2E and Supplementary Table 1), while different metabolites in comparison between the Rb1 and model groups enriched in glycerophospholipid metabolism, linoleic acid metabolism and alpha-linolenic acid metabolism pathway (Figure 2F). Phosphatidylcholine, diacylglycerol and ceramide were the main enriched metabolites in the glycerophospholipid, linoleic acid and alpha-linolenic metabolism pathways, respectively.

In the negative ion model (NEG), 2734 peaks and 1348 metabolites remained, 401 metabolites significantly differed between the control and model group, while 58 metabolites significantly differed between the model and Rb1 group. The same difference trend obtained with the POS model is illustrated in Supplementary Figure 2A, 2B, and the original permutation test of the OPLS-DA model well explains the difference (Supplementary Figure 2C, 2D). KEGG and MetaboAnalyst revealed that glycerophospholipid metabolism, linoleic acid metabolism, alpha-Linolenic acid metabolism, and glycosylphosphatidylinositol (GPI)-anchor biosynthesis were the main enriched pathways when comparing the control and model groups (Supplementary Figure 2E), while glycerophospholipid metabolism, linoleic acid metabolism, alpha-linolenic acid metabolism and arachidonic acid metabolism were the main enriched pathways when comparing the Rb1 and model groups (Supplementary Figure 2F). The main enriched metabolites in the NEG model were the same as in the POS model.

### Characteristics of transcriptome sequencing and differential expression of transcripts

**Figure 3:**
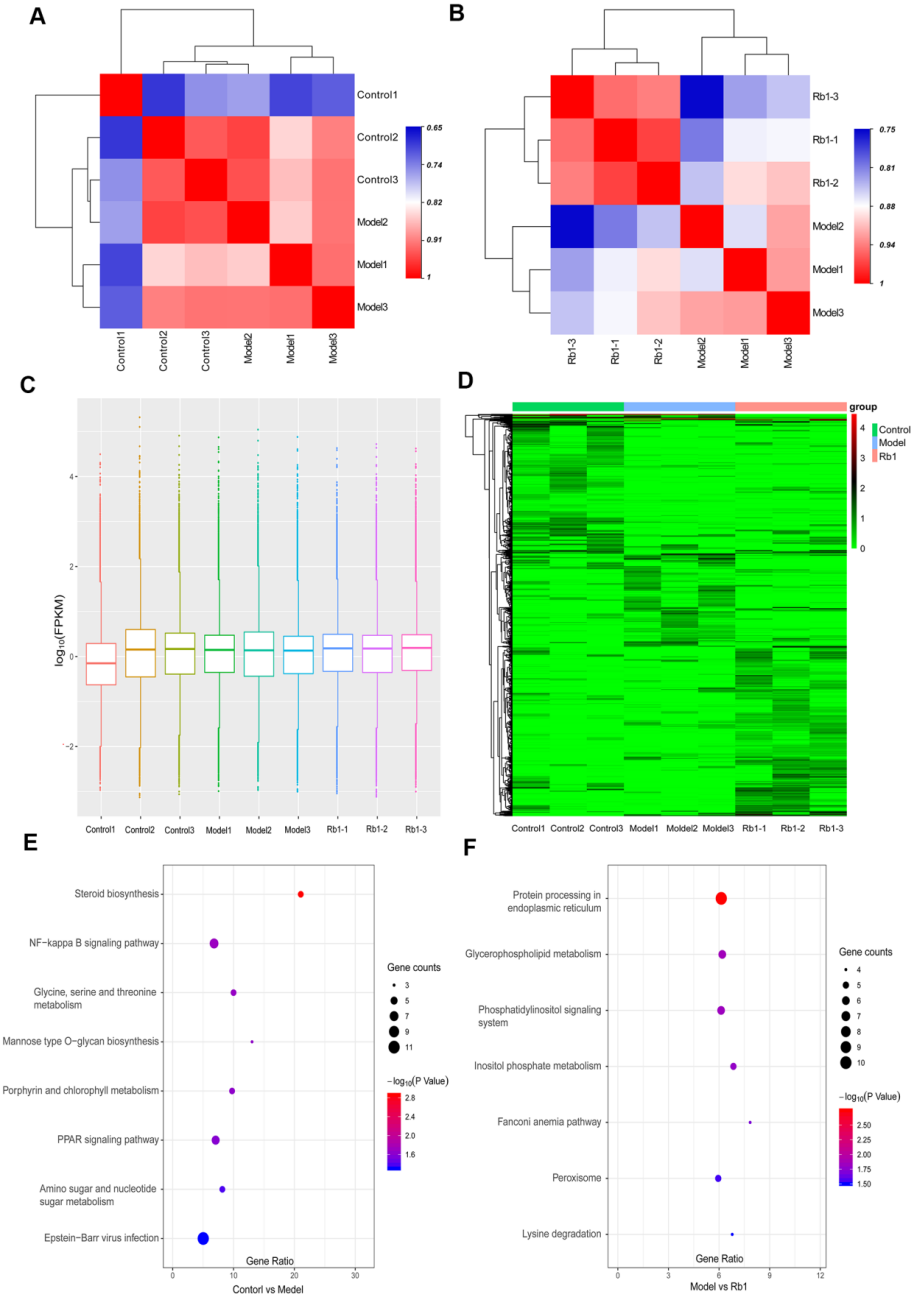
**Characteristics of transcriptomics.** (**A**) Heat map of intersample correlation between model and control group. The correlation was evaluated by Pearson’s correlation coefficient, different color represents differences among different samples. (**B**) Heat map of intersample correlation between Rb1 and model group. (**C**) FPKM distribution boxes of transcripts in each sample. Y axis is shown in log_10_ scale, X axis one sample per region. (**D**) Clustering map of differential genes. The depth of the color represents the level of gene expression. (**E**) KEGG pathway analysis of differentially expressed genes in control group versus model group. Gene ratio represents enrichment factor, bubble scale represents number of different genes, depth of bubble color represents P value. (**F**) KEGG pathway analysis of differentially expressed genes in Rb1 group versus model group.

A total of 866.89 million total raw reads were obtained from the library, and 798.38 million (87.8%) clean reads with an average length of 144.67 nucleotides were screened for subsequent analysis. A total of 75.98% of clean reads mapped to the reference genome. We identified 118,009 transcripts, including 82,038 lncRNAs, 34,641 mRNAs, 418 circRNAs and 912 other RNAs. Only 8.64% of transcripts were known, and most assembled transcripts were novel. Fragments per kilobase of exon per million mapped reads (FPKM) were used to normalize the expression of transcripts. With replicate expression, a heat map was used to distinguish sample groups, and the differences between the control and model groups and between the model and Rb1 groups were distinct (Figure 3A, 3B). The average expression in the 9 samples was nearly 1.0 FPKM (Figure 3C). A total of 510 transcripts were differentially expressed between the model and control group, while 766 transcripts were differentially expressed between the Rb1 and the model group. The significant differences in transcript expression were depicted with clustering analysis (Figure 3D). GO analysis revealed that small molecule metabolic process, cellular carbohydrate metabolic process, regulation of lipid catabolic process, and L-serine metabolic process were the main enriched terms when comparing the model group with the control group (Supplementary Table 2). DNA metabolic process, DNA repair and alpha-amino acid metabolic process were the main enriched terms when comparing the Rb1 and model groups (Supplementary Table 3). The KEGG pathway analysis indicated that glycine, serine and threonine metabolism, mannose type O-glycan biosynthesis, amino sugar and nucleotide sugar metabolism, steroid biosynthesis and the PPAR signaling pathway are involved in the establishment of the high lipid model (Figure 3E). Rb1 may participate in lipid regulation by affecting lysine degradation, inositol phosphate metabolism, and glycerophospholipid metabolism (Figure 3F).

### Comprehensive multiomics analysis reveals that Rb1 affects glycerophospholipid metabolism

**Figure 4:**
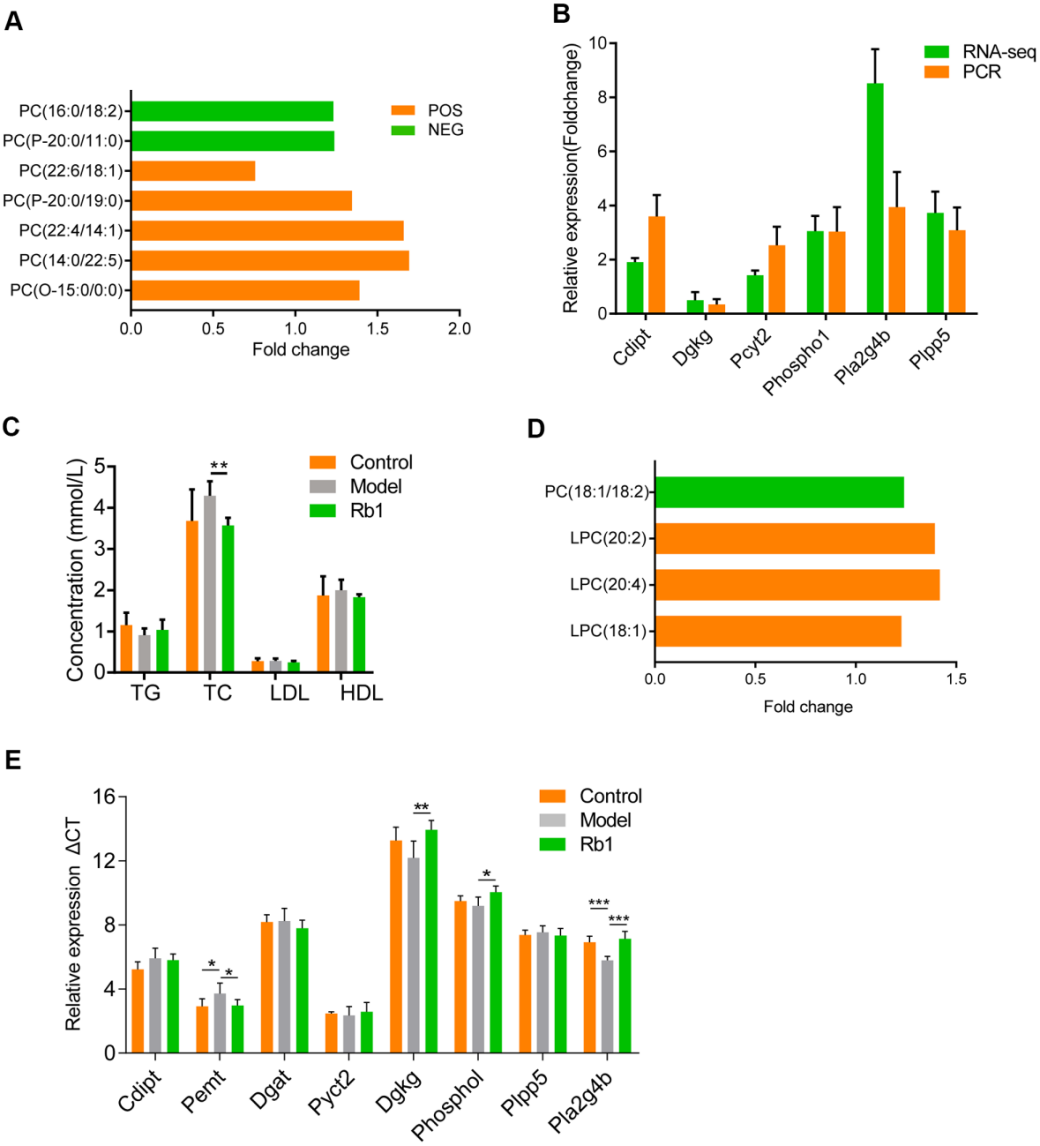
**Comprehensive analysis of microbiology, lipidomics and transcriptomics.** (**A**) Lipidomics showed content of PC increase in normal mice of Rb1 group comparing with that of Model group. Y axis is shown in foldchange, X axis shows different electron cut-off points of PC. All the metabolites with VIP >1 and P <0.05.PC, phosphatidylcholine. POS, positive ion model. NEG, negative ion model. (**B**) RNA-seq and qRT-PCR showed expression of RNAs associated with glycerophospholipid metabolism. RNA-seq was performed once, PCR were repeated thrice, n=3, data was shown in mean +/- SD. (**C**) Serum level of hyperlipidemia associated index showed Rb1 reduced total cholesterol level. TG, triglycerides; TC: total cholesterol; LDL, low density lipoprotein; HDL, high density lipoprotein;** represents P<0.01, n=6. (**D**) Lipidomics showed content of PC increase in fecal transplanted germ-free mice of Rb1 group comparing with that of Model group. Y axis is shown in foldchange, X axis shows different electron cut-off points of PC. All the metabolites with VIP >1 and P <0.05.PC, phosphatidylcholine. (**E**) QRT-PCR showed that Pmet increased and Dgkg, Phospho1, Pla4g4b decreased in fecal transplanted germ-free mice of Rb1 group. This meant Rb1 increased synthesis and decelerate decomposition of phosphatidylcholine.

We found that phosphatidylcholine levels differed between the control and model groups and between the Rb1 and model groups in both the POS and NEG model. When mice were fed Rb1, the level of phosphatidylcholine increased (Figure 4A). Phosphatidylcholine is a key metabolite in the glycerophospholipid metabolism pathway, and its levels in the liver were affected by related genes. Using RNA-seq and qRT-PCR with liver tissue from the Rb1 and model groups, we found that *Cdipt, Dgkg, Pcyt2, Phospho1, Pla2g4b* and *Plpp5* were enriched in glycerophospholipid metabolism pathway, and they were both dysregulated (Figure 4B). This means that Rb1 affects key enzymes involved in regulating the glycerophospholipid metabolic pathway. *Dgkg*, *Phospho1* and *Pla2g4b* regulated the level of phosphatidylcholine, which indicates that Rb1 increases level of phosphatidylcholine by modulating the activities of enzymes in the glycerophospholipid metabolic pathway. In addition, comparison of the microbiomes in the Rb1 and model groups revealed that Rb1 alters ether lipid and glycerolipid metabolism in the mouse gut. These two pathways are co-related to glycerophospholipid metabolism. We also found that TC levels were lower in model mice fed Rb1 (Table 1).

**Figure 5:**
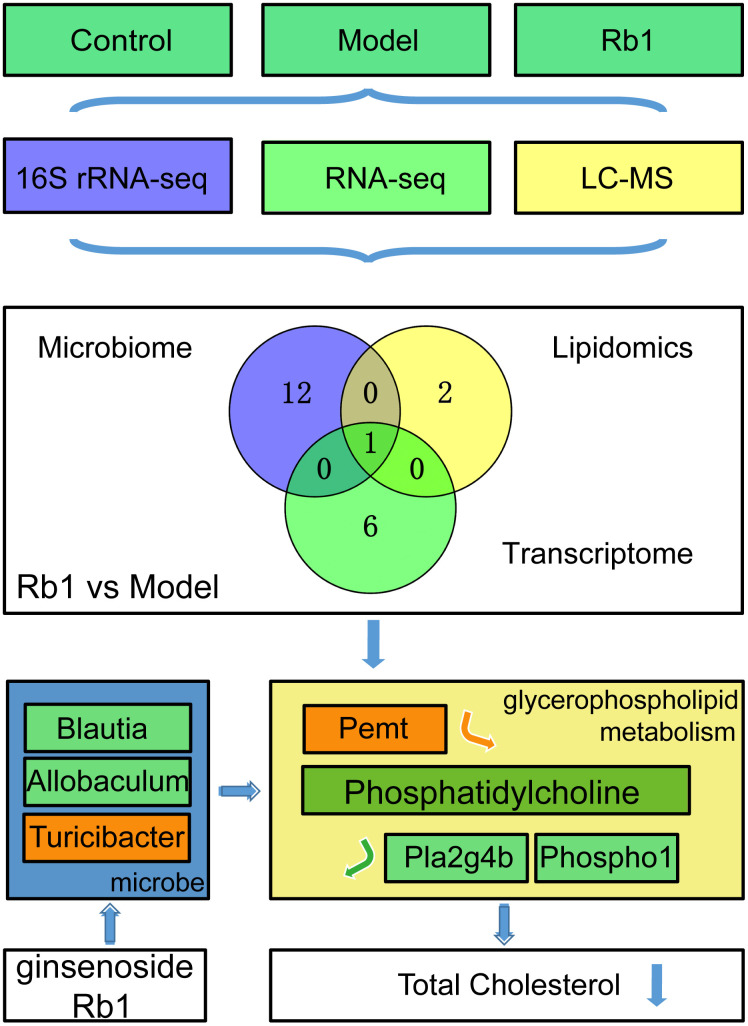
**RB1 increase phosphatidylcholine in glycerophospholipid metabolism to prevent hyperlipidemia.** Through multi-omics analysis, we found Glycerophospholipid metabolism was a common pathway between Rb1 and model group. By using germ-free mice, we found Rb1 increased the abundance of *Turicibacter* and decreased the abundance of *Blautia* and *Allobaculum* to increase content of phosphatidylcholine by raising *Pemt* and reducing *Pla2g4b* and *Phospho1*, and resulting the downregulation of total cholesterol to play a prevention role in hyperlipideamia. Notes: The green box in mocrobe and metabolism box represents the content reduced, the orange box represents the content increased. *Pemt* promotes synthesis of phosphatidylcholine, *Pla2g4b* and *Phospho1* accelerate decomposition of phosphatidylcholine. Notes: Experiments were repeated twice, data was shown in mean +/- SD, * represent P<0.05, ** represent P<0.01, *** represent P<0.001.

Based on the results summarized above, we predicted that to suppress hyperlipidemia, Rb1 modulates gut microbiota to increase the level of phosphatidylcholine in glycerophospholipid metabolism, decreasing TC. To test the idea, three groups of germ-free mice were administered by gavage feces from control, model and Rb1 mice. We subsequently found that serum TC levels were significantly reduced in mice receiving feces from the Rb1 mice (Figure 4C), while phosphatidylcholine was increased (Figure 4D). In addition, levels of *Pemt* mRNA were increased and levels of *Phospho1* and *Pla2g4b* were decreased (Figure 4E). These findings support our prediction that, in these mice, Rb1 modulates gut microbiota to regulate the synthesis and decomposition of phosphatidylcholine in glycerophospholipid metabolism, which in turn decreases serum TC, suppressing hyperlipidemia (Figure 5).

## DISCUSSION

Abnormal cholesterol production, transport and metabolism are important causes of hyperlipidemia [15], as cholesterol homeostasis plays a key role in determining blood lipid level [16]. Recent studies investigating the influence of intestinal microbiome composition on cholesterol metabolism indicate that gut-microbiota-targeted therapy may be a useful strategy for treating hyperlipidemia [17]. Drugs such as statins [18, 19] theabrownin [20] and PTF [21] as well as polyunsaturated fatty acids from microalgae Spirulina platensis [22], which modulate gut microbiota, are under investigation for the treatment of hyperlipidemia.

The results the present study show that while serum levels of TG, TC and LDL-C are significantly elevated in hyperlipidemic mice, administration of ginsenoside Rb1 significantly decreased serum HDL-C levels and can ameliorate lipid metabolic disorders. Statistical analysis of annotated species revealed that *Blautia* and *Allobaculum* were increased in the hyperlipidemic model mice as compared to control mice but were decreased in Rb1 mice compared to model mice, while the *Turicibacter* profile was the opposite. *Blautia* are fatty acid-producing bacteria associated with atherosclerosis [23]. TG, TC, and LDL all correlated positively with the relative abundance of genera of *Blautia* [24]. In addition, Meng [25] found that *Allobaculum* affects lipid metabolism, and Tang [26] reported that *Blautia* and *Allobaculum* exhibit significant positive correlations with TG, TC, LDL-C, IL-6, IL-1β, TNF-α and body weight, and a negative correlation with HDL-C. These findings are all consistent with the results of the present study and suggest that too much *Blautia* and *Allobaculum* could potentially cause hyperlipidemia. *Turicibacter* is a genus associated with disorders in lipid metabolism and has significant anti-obesity effects [27]. Zheng [28] found that *Turicibacter* correlated positively with HDL-C level. The results summarized above indicate that these bacteria could suppress hyperlipidemia, which is consistent with the results of the present experiment. KEGG analysis showed that glycerophospholipid metabolism is a differentially enriched between the Rb1 and model group. We therefore suggested that ginsenoside Rb1 exerts a lipid-lowering effect that prevent hyperlipidemia by affecting gut microbes and in turn glycerophospholipid metabolism.

Of the thousands of molecules in a living cell, PC has been studied as one of the most fundamental and important. A vital component of cell membranes, it is the most abundant of what are considered “essential phospholipids.” Ingestion of PC has a number of benefits, including improved memory, better intestinal and skin health, improved lipid metabolism and weight loss. Focusing on its lipid-lowering effects, PC helps cholesterol to dissolve more easily, which would suppress the buildup of cholesterol within arteries, thereby reducing the risk of atherosclerosis. In fact, PC helps lower in tissues throughout the body [29], making PC metabolism a crucial factor for decreasing bodily lipid levels.

In our study, lipidomics analysis revealed that linoleic acid metabolism, alpha-linolenic acid metabolism, glycerophospholipid metabolism and arachidonic acid metabolism were all differentially enriched between the model and ginsenoside Rb1 groups. In the transcriptomic analysis, the KEGG enrichment results showed that inositol phosphate metabolism, phosphatidylinositol signaling system and glycerophospholipid metabolism were the most prominent pathways associated with genes differentially expressed between the model and Rb1 groups. The expression of genes involved in PC biosynthesis, including *PEMT*, were upregulated, while expression of metabolic genes, including *Pla2g4b*, *phospho1*, and *Dgkg* were downregulated. Phosphatidylethanolamine (PE) N-methyltransferase (PEMT) catalyzes the synthesis of PC through successive methylation of the amino headgroup of PE [30, 31]. Dgkg encodes an enzyme that is a member of the type I subfamily of diacylglycerol kinases, which are involved in lipid metabolism [32]. PC is decomposed into acetylcholine or lysophosphatidylglycreol by Phospho1 and Dgkg [33]. Integrated microbiology, lipidomics and transcriptome analyses showed that glycerophospholipid metabolism is a common pathway. The key metabolite of these pathways is PC. Previous studies showed that in addition to being a major component of cell membranes, PC is an important signaling molecule involved in atherosclerosis [34] PC enhances the assembly of VLDL in hepatocytes and the production of bile acids in the liver, thereby facilitating lipid export and regulating lipid metabolism [35, 36]. The results of the present study suggest that ginsenoside Rb1 affects hyperlipidemia by regulating gut bacteria and increasing the production of PC.

In summary, we have shown the therapeutic effects of Rb1 on hyperlipidemic rats. The comprehensive multiomics analysis was valuable for elaborating the functional mechanism by which Rb1 reduces hyperlipidemia. It can be concluded that Rb1 acts by regulating intestinal flora to increase their PC content and reduce cholesterol. Ginsenoside Rb1 is thus a promising candidate for reversing aberrant lipid metabolism in metabolic disorders.

## MATERIALS AND METHODS

### Mice and samples

The Ethics Committee of Liaoning University of Traditional Chinese Medicine approved and supervised the research protocol. Twenty-one mice were divided into 3 equal groups: a control group fed a regular diet, a model group fed a high-fat diet for 10 weeks, and a Rb1 group fed a high-fat diet and Rb1 (200 mg·kg^-1^·d^-1^). Stool, serum and hepatic tissues were collected. The samples were stored at -80° C immediately after separation.

### RNA extraction

Total RNA was extracted from hepatic tissues with TRIzol (Invitrogen, USA) according to the manufacturer’s instructions. The RNA concentration and purity were assessed with an Agilent 2100 Bioanalyzer (Agilent Technologies, USA) and an RNA 6000 Nano LabChip Kit (Agilent Technologies, USA).

### Microbiome sequencing process and analysis

Stool samples from 3 mice in the control group, 5 mice in the model group and 5 mice in the Rb1 group were used for microbiome sequencing. After the amplification of the V3 and V4 regions, a library was quantified and pooled, after which Agilent 2100 Bioanalyzer was used to evaluate the library. Microbiome rRNA sequencing was performed with a MiSeq instrument. TrimGalore was used remove base pairs of less than 20 base pairs and the possible adapter sequences. Then short sequences less than 100 bp in length were removed, and FLASH2 was used to obtain the merge sequence and remove low-quality sequence. Mother was used to research and remove the primers in the sequence. Usearch was used to remove sequences with a total base error rate greater than 2 and sequences with lengths less than 100 bp in order to get clean, high-quality, reliable reads for subsequent bioinformatics analysis. Operational taxonomic units (OTUs) were used to measure the similarity between two sequences, and the threshold was set at 97%. OTU taxonomic annotation was obtained after sequence mapping to a database. TaxonTree in the R software package was used to reveal the genus distribution of each species in all samples; only relative abundances greater than 1% are shown. Based on the OTU abundance table, variance decomposition performed through principal component analysis (PCA) using R software was determined to reflect the differences between samples on a two-dimensional graph. The PICRUSt analysis tool was used to predict and analyze species functions based on amplicon sequencing data.

### Lipidomics experiment and analysis

For non-target lipidomics, all 21 mice were used for the lipidomics experiment. Serum samples were thawed at 4° C, after which 30-μl aliquots were mixed with CH2Cl2-CH3OH (containing 0.1 g/L 2,6-tert butyl-4-methyphenol, 0° C), and 600 μL of mixed solvent was added. The mixture was then vortexed, incubated at room temperature and, after 200 μL of water were added, centrifuged. The organic phase was removed, dried with nitrogen, mixed with acetonitrile-isopropanol (CH3CN-IPA, 1:1, V/V), redissolved in 500 μL of mixed solvent, and centrifuged at 10,000 rpm for 10 min. The supernatant was then collected, and 10 μL was collected from each sample and mixed thoroughly to obtain the QC samples. Ultrahigh-performance liquid tandem chromatography quadrupole time of flight mass spectrometry (UHPLC-QTOFMS) was used for metabolite detection in the 3 groups. Data were obtained in positive ion mode (POS) and negative ion mode (NEG). The three-dimensional data, including the peak number, sample name, and normalized peak area were imported into the SIMCA14.1 software package (V14.1, Sartorius Stedim Data Analytics AB, Umea, Sweden) for orthogonal projections to latent structures-discriminate analysis (OPLS-DA). A permutation test was performed to further validate the OPLS-DA model. The first principal component of variable importance in the projection (VIP) was obtained from the OPLS-DA model. The criteria VIP >1 and P value< 0.05 (assessed by Student’s t-test) were used to screen differential metabolites between two comparison groups. KEGG (http://www.genome.jp/kegg/) and MetaboAnalyst (http://www.metaboanalyst.ca/) were utilized to search for the pathways of metabolites.

For target lipidomics, 10 μL of the samples were mixed with 190 μL water, after which 480 μL of extract solution (MTBE:MeOH = 5:1) containing an internal standard was added. The samples were sonicated, centrifuged, and the supernatants were transferred for drying. The dried samples were reconstituted in resuspension buffer (DCM:MeOH:H2O= 60:30:4.5) by sonication. The samples were then centrifuged for LC/MS analysis. UHPLC separation was carried out using a SCIEX ExionLC series UHPLC System. An AB Sciex QTrap 6500+ mass spectrometer was applied for assay development. Typical ion source parameters were: IonSpray Voltage: +5500/-4500 V, Curtain Gas: 40 psi, Temperature: 350° C, Ion Source Gas 1:50 psi, Ion Source Gas 2: 50 psi, DP: ±80V.

### Transcriptomic sequencing process and analysis

Hepatic tissues from 3 mice from each group were used for whole transcriptome analysis. Libraries were constructed, and deep sequencing was performed using a HiSeq X instrument. The read counts of each transcript were normalized by FPKM. A fold-change >2 and P<0.05 between two samples were the criteria used to identify differentially expressed transcripts. GO (http://www.geneontology.org) and KEGG analyses were performed to construct annotations for differentially expressed transcripts. P values were used to test the reliability of the analysis.

### Quantitative real-time PCR validation

qRT-PCR was performed according to the manufacturer’s instructions. β-Actin was used as an endogenous reference. The 2^−∆∆CT^ method was used to calculate relative RNA expression levels. Student’s t-tests were used to assess differences; P<0.05 was considered significant. The primer sequences are shown in Supplementary Table 4.

### Germ-free mice

Eighteen 6-week-old germ free (GF) male mice were fed in a sterile isolator in the experimental animal center at the Shanghai Shrek Experimental Animal Co., Ltd. The temperature was maintained at 20-22° C, and the humidity was maintained at 50-60%. The water, feed and bedding materials were irradiated with 50 kGy radiation, and the drinking bottle and cage were sterilized under high temperature and pressure at 121° C for 60 min. The day/night cycle was 12/12.

### Blood lipid detection

The night before the material was taken, the mice fasted and couldn't help water. On the day of sampling, the mice were anesthetized using 1% pentobarbital (0.2 ml/mouse). Blood was collected from abdominal aorta and centrifuged at 3000 rpm for 30 min, after which the serum was collected and stored at -80° C. Lipid detection was then carried out according to the instructions of the biochemical kit (HDL-C, LDL-C, TG, TC; FuJIFILm Corporation) and the operation process of the instrument (automatic biochemical analyzer; FuJIFILm FDC-7000I).

### Hematoxylin-eosin staining

After fixing samples in 4% paraformaldehyde, they were dehydrated through an ethanol gradient, embedded in paraffin in the embedding machine, and cut into 4-μm-thick sections. For HE staining, the sections were deparaffinized with xylene, hydrated through an ethanol gradient, stained with HE, dehydrated in alcohol and xylene, and mounted on slides. Images were acquired using a Nikon Eclipse CI microscope.

### Oil red O staining

The tissues were dehydrated in 15% and 30% sucrose and then embedded in paraffin to prepare 8- to 10-μm-thick frozen sections. The frozen sections were fixed, soaked in oil red O dye, counterstained with hematoxylin and sealed in glycerin gelatin. Images were acquired with a Nikon E100 microscope.

## Supplementary Material

Supplementary Figures

Supplementary Table 1

Supplementary Table 2

Supplementary Table 3

Supplementary Table 4

## References

[1] Wu J, Cheng X, Qiu L, Xu T, Zhu G, Han J, Xia L, Qin X, Cheng Q, Liu Q. Prevalence and clustering of major cardiovascular risk factors in China: a recent cross-sectional survey. Medicine (Baltimore). 2016; 95:e2712. 10.1097/MD.0000000000002712 26962771 PMC4998852

[2] Vishram JK. Prognostic interactions between cardiovascular risk factors. Dan Med J. 2014; 61:B4892. 25123126

[3] Liu C, Hu MY, Zhang M, Li F, Li J, Zhang J, Li Y, Guo HF, Xu P, Liu L, Liu XD. Association of GLP-1 secretion with anti-hyperlipidemic effect of ginsenosides in high-fat diet fed rats. Metabolism. 2014; 63:1342–51. 10.1016/j.metabol.2014.06.015 25060691

[4] Park KH, Shin HJ, Song YB, Hyun HC, Cho HJ, Ham HS, Yoo YB, Ko YC, Jun WT, Park HJ. Possible role of ginsenoside Rb1 on regulation of rat liver triglycerides. Biol Pharm Bull. 2002; 25:457–60. 10.1248/bpb.25.457 11995924

[5] Ma H, Zhang B, Hu Y, Wang J, Liu J, Qin R, Lv S, Wang S. Correlation analysis of intestinal redox state with the gut microbiota reveals the positive intervention of tea polyphenols on hyperlipidemia in high fat diet fed mice. J Agric Food Chem. 2019; 67:7325–35. 10.1021/acs.jafc.9b02211 31184120

[6] Wang Y, Tong Q, Shou JW, Zhao ZX, Li XY, Zhang XF, Ma SR, He CY, Lin Y, Wen BY, Guo F, Fu J, Jiang JD. Gut microbiota-mediated personalized treatment of hyperlipidemia using berberine. Theranostics. 2017; 7:2443–51. 10.7150/thno.18290 28744326 PMC5525748

[7] Zhu Y, Li T, Din AU, Hassan A, Wang Y, Wang G. Beneficial effects of enterococcus faecalis in hypercholesterolemic mice on cholesterol transportation and gut microbiota. Appl Microbiol Biotechnol. 2019; 103:3181–91. 10.1007/s00253-019-09681-7 30783721

[8] Chiu HF, Chen YJ, Lu YY, Han YC, Shen YC, Venkatakrishnan K, Wang CK. Regulatory efficacy of fermented plant extract on the intestinal microflora and lipid profile in mildly hypercholesterolemic individuals. J Food Drug Anal. 2017; 25:819–27. 10.1016/j.jfda.2016.10.008 28987358 PMC9328888

[9] Wang K, Liao M, Zhou N, Bao L, Ma K, Zheng Z, Wang Y, Liu C, Wang W, Wang J, Liu SJ, Liu H. Parabacteroides distasonis alleviates obesity and metabolic dysfunctions via production of succinate and secondary bile acids. Cell Rep. 2019; 26:222–35.e5. 10.1016/j.celrep.2018.12.028 30605678

[10] Schroeder BO, Bäckhed F. Signals from the gut microbiota to distant organs in physiology and disease. Nat Med. 2016; 22:1079–89. 10.1038/nm.4185 27711063

[11] Miao H, Zhao YH, Vaziri ND, Tang DD, Chen H, Chen H, Khazaeli M, Tarbiat-Boldaji M, Hatami L, Zhao YY. Lipidomics biomarkers of diet-induced hyperlipidemia and its treatment with poria cocos. J Agric Food Chem. 2016; 64:969–79. 10.1021/acs.jafc.5b05350 26758241

[12] Tu L, Sun H, Tang M, Zhao J, Zhang Z, Sun X, He S. Red raspberry extract (Rubus idaeus L shrub) intake ameliorates hyperlipidemia in HFD-induced mice through PPAR signaling pathway. Food Chem Toxicol. 2019; 133:110796. 10.1016/j.fct.2019.110796 31472226

[13] Dong WW, Xuan FL, Zhong FL, Jiang J, Wu S, Li D, Quan LH. Comparative Analysis of the Rats' Gut Microbiota Composition in Animals with Different Ginsenosides Metabolizing Activity. J Agric Food Chem. 2017; 65:327–37. 10.1021/acs.jafc.6b04848 28025886

[14] Li N, Zhou L, Li W, Liu Y, Wang J, He P. Protective effects of ginsenosides Rg1 and Rb1 on an Alzheimer’s disease mouse model: a metabolomics study. J Chromatogr B Analyt Technol Biomed Life Sci. 2015; 985:54–61. 10.1016/j.jchromb.2015.01.016 25660715

[15] Yang D, Hu C, Deng X, Bai Y, Cao H, Guo J, Su Z. Therapeutic effect of chitooligosaccharide tablets on lipids in high-fat diets induced hyperlipidemic rats. Molecules. 2019; 24:514. 10.3390/molecules24030514 30709014 PMC6385166

[16] El-Tantawy WH, Temraz A. Natural products for controlling hyperlipidemia: review. Arch Physiol Biochem. 2019; 125:128–35. 10.1080/13813455.2018.1441315 29457523

[17] Catry E, Pachikian BD, Salazar N, Neyrinck AM, Cani PD, Delzenne NM. Ezetimibe and simvastatin modulate gut microbiota and expression of genes related to cholesterol metabolism. Life Sci. 2015; 132:77–84. 10.1016/j.lfs.2015.04.004 25916803

[18] Khan TJ, Ahmed YM, Zamzami MA, Siddiqui AM, Khan I, Baothman OA, Mehanna MG, Kuerban A, Kaleemuddin M, Yasir M. Atorvastatin treatment modulates the gut microbiota of the hypercholesterolemic patients. OMICS. 2018; 22:154–63. 10.1089/omi.2017.0130 29432061

[19] Kim J, Lee H, An J, Song Y, Lee CK, Kim K, Kong H. Alterations in gut microbiota by statin therapy and possible intermediate effects on hyperglycemia and hyperlipidemia. Front Microbiol. 2019; 10:1947. 10.3389/fmicb.2019.01947 31551944 PMC6736992

[20] Huang F, Zheng X, Ma X, Jiang R, Zhou W, Zhou S, Zhang Y, Lei S, Wang S, Kuang J, Han X, Wei M, You Y, et al. Theabrownin from Pu-erh tea attenuates hypercholesterolemia via modulation of gut microbiota and bile acid metabolism. Nat Commun. 2019; 10:4971. 10.1038/s41467-019-12896-x 31672964 PMC6823360

[21] Wu C, Tian Y, Yu J, Zhang R, Zhang X, Guo P. The pandanus tectorius fruit extract (PTF) modulates the gut microbiota and exerts anti-hyperlipidaemic effects. Phytomedicine. 2019; 58:152863. 10.1016/j.phymed.2019.152863 30836215

[22] Li TT, Tong AJ, Liu YY, Huang ZR, Wan XZ, Pan YY, Jia RB, Liu B, Chen XH, Zhao C. Polyunsaturated fatty acids from microalgae spirulina platensis modulates lipid metabolism disorders and gut microbiota in high-fat diet rats. Food Chem Toxicol. 2019; 131:110558. 10.1016/j.fct.2019.06.005 31175915

[23] Yue C, Li M, Li J, Han X, Zhu H, Yu G, Cheng J. Medium-, long- and medium-chain-type structured lipids ameliorate high-fat diet-induced atherosclerosis by regulating inflammation, adipogenesis, and gut microbiota in ApoE-/- mice. Food Funct. 2020; 11:5142–55. 10.1039/d0fo01006e 32432606

[24] Li X, Xiao Y, Song L, Huang Y, Chu Q, Zhu S, Lu S, Hou L, Li Z, Li J, Xu J, Ren Z. Effect of lactobacillus plantarum HT121 on serum lipid profile, gut microbiota, and liver transcriptome and metabolomics in a high-cholesterol diet-induced hypercholesterolemia rat model. Nutrition. 2020; 79:110966. 10.1016/j.nut.2020.110966 32942130

[25] Meng XL, Li S, Qin CB, Zhu ZX, Hu WP, Yang LP, Lu RH, Li WJ, Nie GX. Intestinal microbiota and lipid metabolism responses in the common carp (Cyprinus carpio L.) following copper exposure. Ecotoxicol Environ Saf. 2018; 160:257–64. 10.1016/j.ecoenv.2018.05.050 29852428

[26] Tang W, Yao X, Xia F, Yang M, Chen Z, Zhou B, Liu Q. Modulation of the gut microbiota in rats by hugan qingzhi tablets during the treatment of high-fat-diet-induced nonalcoholic fatty liver disease. Oxid Med Cell Longev. 2018; 2018:7261619. 10.1155/2018/7261619 30671174 PMC6323444

[27] Wu M, Yang S, Wang S, Cao Y, Zhao R, Li X, Xing Y, Liu L. Effect of berberine on atherosclerosis and gut microbiota modulation and their correlation in high-fat diet-fed ApoE-/- mice. Front Pharmacol. 2020; 11:223. 10.3389/fphar.2020.00223 32231564 PMC7083141

[28] Zheng B, Wang T, Wang H, Chen L, Zhou Z. Studies on nutritional intervention of rice starch- oleic acid complex (resistant starch type V) in rats fed by high-fat diet. Carbohydr Polym. 2020; 246:116637. 10.1016/j.carbpol.2020.116637 32747272

[29] Breidigan JM, Krzyzanowski N, Liu Y, Porcar L, Perez-Salas U. Influence of the membrane environment on cholesterol transfer. J Lipid Res. 2017; 58:2255–63. 10.1194/jlr.M077909 29046341 PMC5711489

[30] Shields DJ, Lehner R, Agellon LB, Vance DE. Membrane topography of human phosphatidylethanolamine N-methyltransferase. J Biol Chem. 2003; 278:2956–62. 10.1074/jbc.M210904200 12431977

[31] Hörl G, Wagner A, Cole LK, Malli R, Reicher H, Kotzbeck P, Köfeler H, Höfler G, Frank S, Bogner-Strauss JG, Sattler W, Vance DE, Steyrer E. Sequential synthesis and methylation of phosphatidylethanolamine promote lipid droplet biosynthesis and stability in tissue culture and *in vivo*. J Biol Chem. 2011; 286:17338–50. 10.1074/jbc.M111.234534 21454708 PMC3089575

[32] Guo Z, Jia J, Yao M, Kang J, Wang Y, Yan X, Zhang L, Lv Q, Chen X, Lu F. Diacylglycerol kinase γ predicts prognosis and functions as a tumor suppressor by negatively regulating glucose transporter 1 in hepatocellular carcinoma. Exp Cell Res. 2018; 373:211–20. 10.1016/j.yexcr.2018.11.001 30399372

[33] Lagace TA, Ridgway ND. The role of phospholipids in the biological activity and structure of the endoplasmic reticulum. Biochim Biophys Acta. 2013; 1833:2499–510. 10.1016/j.bbamcr.2013.05.018 23711956

[34] Kougias P, Chai H, Lin PH, Lumsden AB, Yao Q, Chen C. Lysophosphatidylcholine and secretory phospholipase A2 in vascular disease: mediators of endothelial dysfunction and atherosclerosis. Med Sci Monit. 2006; 12:RA5–16. 16369478

[35] Christensen KE, Wu Q, Wang X, Deng L, Caudill MA, Rozen R. Steatosis in mice is associated with gender, folate intake, and expression of genes of one-carbon metabolism. J Nutr. 2010; 140:1736–41. 10.3945/jn.110.124917 20724492

[36] Zhao R, Matherly LH, Goldman ID. Membrane transporters and folate homeostasis: intestinal absorption and transport into systemic compartments and tissues. Expert Rev Mol Med. 2009; 11:e4. 10.1017/S1462399409000969 19173758 PMC3770294

