# Supplementary material for: Comprehensive multiomics analysis of the effect of ginsenoside Rb1 on hyperlipidemia
Source: Aging (Albany NY). 2021 Mar 19;13(7):9732–47. doi: 10.18632/aging.202728 (PMC8064217; doi:10.18632/aging.202728)
Supplement: Supplementary Figures [file aging-13-202728-s001.pdf]

SUPPLEMENTARY FIGURES

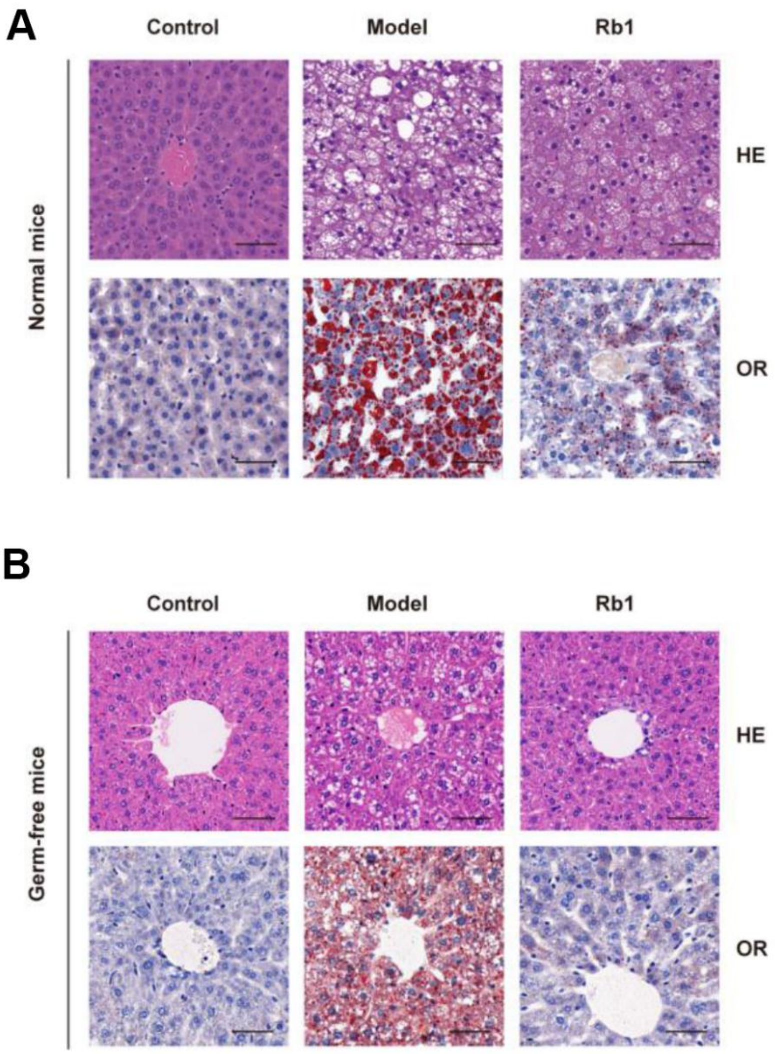

Supplementary Figure 1. Histopathological examination of liver tissue in hyperlipidemia rats. (**A**) Histopathological examination of liver in normal mice. HE and Oil red staining showed that compared with the normal group, the content of lipid droplets in liver tissue of rats from model group was significantly increased, and when model group fed with ginsenoside Rb1 content of lipid droplets significantly decreased. (**B**) Histopathological examination of liver in fecal transplanted germ-free mice. HE and Oil red staining showed that compared with the normal group, the content of lipid droplets in liver tissue of rats from model group was significantly increased, and when model group fed with ginsenoside Rb1 content of lipid droplets significantly decreased. The resolution was 40×.

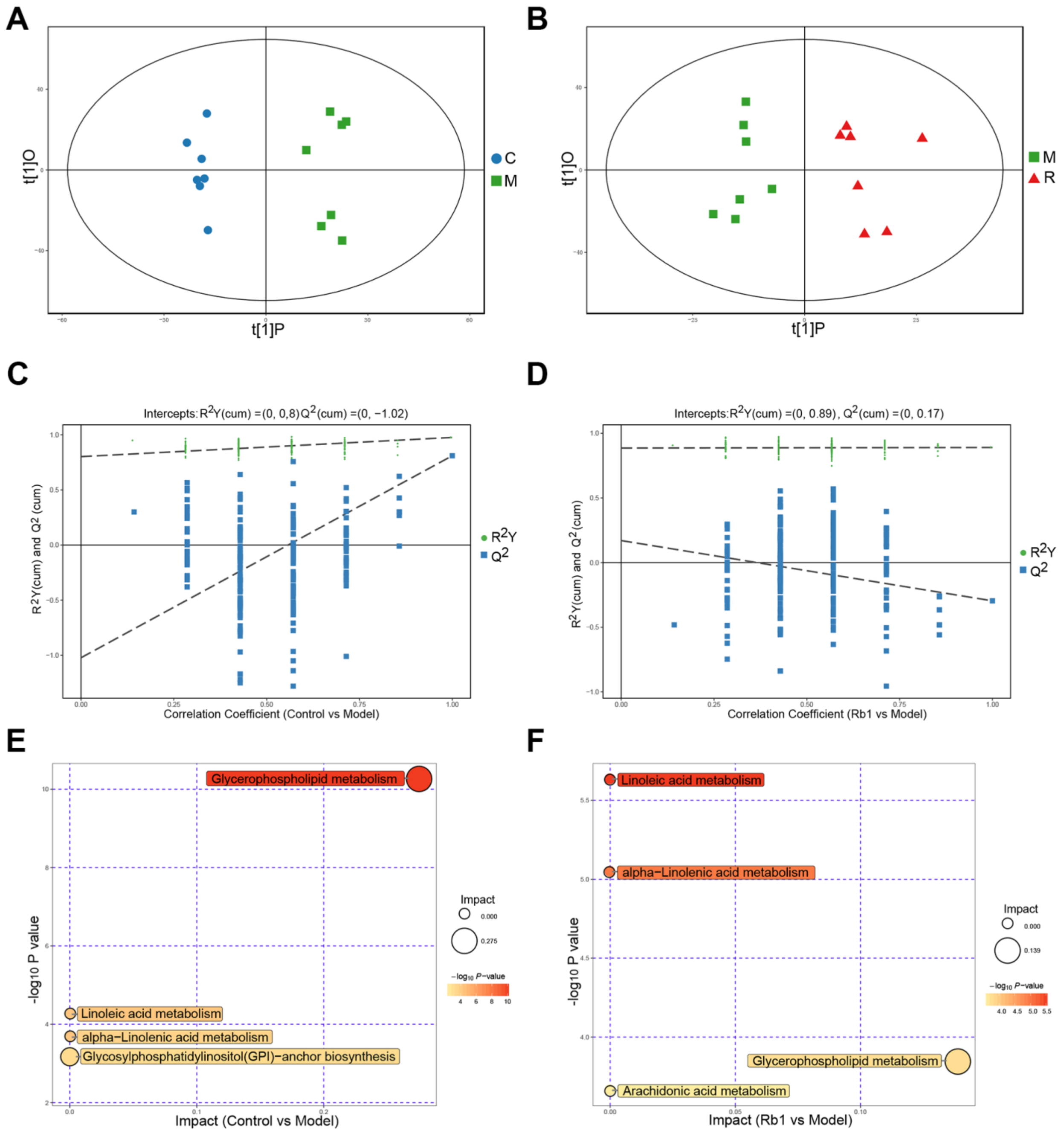

Supplementary Figure 2. Characteristics of lipidomics in negative ion model. (**A**) Score scatter plot of OPLS-DA model for group C vs M in NEG model. The X axis t [1] P denotes the predicted principal component score of the first principal component, the Y axis t [1] O denotes the predicted principal component score of the first principal component, the Y axis t [1] O denotes the orthogonal principal component scores. The two groups of samples are very distinct in this model. C, control group M, model group. NEG. M, model group. NEG, negative ion model. (**B**) Scores scatter plot of OPLS-DA model for group M vs R in NEG. M, model group. R, Rb1 group. NEG, negative ion model (**C**) Permutation test of OPLS-DA model for group Control vs Model in NEG. The X axis represents the replacement retention, the Y axis represents the value of R2Y or Q2, the green dot represents the value of R2Y and the blue square represents the value of Q of the replacement test. The dotted lines represent the regression lines of R Y and Q, respectively. The original model can well explain the difference between the two groups of samples. (**D**) Permutation test of OPLS-DA model for group Rb1 vs Model in NEG. (**E**) Pathway analysis for group Control vs Model in NEG. The bubble size indicate the influencing factor in the topological analysis; the bubble color represent the P value of enrichment analysis. (**F**) Pathway analysis for group Rb1 vs Model in NEG.
