# Supplementary material for: Comprehensive multiomics analysis of the effect of ginsenoside Rb1 on hyperlipidemia
Source: Aging (Albany NY). 2021 Mar 19;13(7):9732–47. doi: 10.18632/aging.202728 (PMC8064217; doi:10.18632/aging.202728)
Supplement: Supplementary Table 4 [file aging-13-202728-s005.pdf]

Supplementary Table 4. The primer sequences.

Gene	Sequence	Product length
Cdipt	Forward primerCCCGGATTGTTTTCGCCATC	144
	Reverse primerCCCCAAATCGGGTTCCTTGA	
Dgkg	Forward primerAGTGGTGGGTCTAGAGGGAG	223
	Reverse primerGAAGCTGCTCTTCTGGGGAG	
Pcyt2	Forward primerGTACAGAGGCTTTGGGGACC	259
	Reverse primerTTCCTGCTCCTTCCTAGGGC	
Phospho1	Forward primerTCTTGGATGTGCCAGCGACC	274
	Reverse primerAGTAGCCCTCCCGATAGGTG	
Pla2g4b	Forward primerGCTCCCTGAGTCTCGCATTT	228
	Reverse primerCGCTTTGTCAGGAGGTCAGT	
Plpp5	Forward primerTACAGCCACAGGGACAAACG	183
	Reverse primerAACATGTGTGTTGGGCAGGA	
